# Production and Characterization of Chitosan–Polyanion Nanoparticles by Polyelectrolyte Complexation Assisted by High-Intensity Sonication for the Modified Release of Methotrexate

**DOI:** 10.3390/ph13010011

**Published:** 2020-01-08

**Authors:** Yhors Ciro, John Rojas, Maria J. Alhajj, Gustavo A. Carabali, Constain H. Salamanca

**Affiliations:** 1 Department of Pharmacy, School of Pharmaceutical and Food Sciences, University of Antioquia, 67 Street No. 53-108, Medellín 050010, Colombia; jrojasca@gmail.com; 2 Laboratorio de Diseño y Formulación de Productos Químicos y Derivados, Departamento de Ciencias Farmacéuticas, Facultad de Ciencias Naturales, Universidad ICESI, Calle 18 No. 122-135, Cali 760035, Colombia; mariajoalhajj@hotmail.com (M.J.A.); carabalo777@hotmail.com (G.A.C.)

**Keywords:** polyelectrolyte complexation, chitosan, poly (maleic acid-*alt*-ethylene), poly (maleic acid-*alt*-octadecene), methotrexate nanoparticles, polyelectrolyte complexation, modified release

## Abstract

A promising strategy to improve the effectivity of anticancer treatment and decrease its side effects is to modulate drug release by using nanoparticulates (NPs) as carriers. In this study, methotrexate-loaded chitosan–polyanion nanoparticles were produced by polyelectrolyte complexation assisted by high-intensity sonication, using several anionic polymers, such as the sodium and potassium salts of poly(maleic acid-*alt*-ethylene) and poly(maleic acid-*alt*-octadecene), here named PAM-2 and PAM-18, respectively. Such NPs were analyzed and characterized according to particle size, polydispersity index, zeta potential and encapsulation efficiency. Likewise, their physical stability was tested at 4 °C and 40 °C in order to evaluate any change in the previously mentioned particle parameters. The in vitro methotrexate release was assessed at a pH of 7.4, which simulated physiological conditions, and the data were fitted to the heuristic models of order one, Higuchi, Peppas–Sahlin and Korsmeyer–Peppas. The results revealed that most of the MTX-chitosan–polyanion NPs have positive zeta potential values, sizes <280 nm and monodisperse populations, except for the NPs formed with PAM-18 polyanions. Further, the NPs showed adequate physical stability, preventing NP–NP aggregation. Likewise, these carriers modified the MTX release by an anomalous mechanism, where the NPs formed with PAM-2 polymer led to a release mechanism controlled by diffusion and relaxation, whereas the NPs formed with PAM-18 led to a mainly diffusion-controlled release mechanism.

## 1. Introduction

Cancer is the second leading cause of death worldwide and a growing issue for public health, especially in developed countries [1,2]. Chemotherapy is the preferred first line treatment for cancer. However, many chemotherapeutic agents have low aqueous solubility and their efficacy is questionable because cancer cells are able to attain resistance, mainly due to efflux pumps [3,4]. Currently, methotrexate (MTX) is widely used as monotherapy or in combination with other anticancer drugs for the treatment of several cancer types, such as brain tumors, primary central nervous system lymphoma, and leptomeningeal metastatic cancer [5,6]. However, the use of MTX is limited due to its poor solubility, non-specific drug delivery and toxic side effects, which leads to disruption of the anticancer treatment for the patient [7]. Nanocarriers, as chemotherapeutic agents, offer novel strategies (i.e., liposomes, polymeric and inorganic nanoparticles, micelles, dendrimers and carbon nanotubes) for targeting cancer cells and tumor zones without causing major damage to the surrounding healthy tissue, and hence, they allow the chemotherapeutic agent to accumulate in tumor zones due to their enhanced permeation and retention effect [8,9]. Chitosan (CH) is a non-toxic, biocompatible, biodegradable, and adsorptive polymer with proven anticancer activity against different cancer cell lines due to disruption of the G1/S phases of the cell cycle and its ability to increase TUNEL-positive cells, which is accompanied by a subtle increase in caspase activity [10]. In turn, this activity depends on the molecular weight and degree of acetylation, as these properties affect the solubility of CH. Therefore, a low molecular weight and acetylation degree in CH causes a more pronounced anticancer effect [11,12]. Thus, multiple nanoparticulate systems have been developed that release MTX in a controlled fashion, and therefore enhancing its apoptotic effect [13,14,15,16].

For the production of chitosan NPs, different methods can be used, such as (i) emulsification by solvent evaporation, (ii) emulsification by solvent diffusion, (iii) coacervation, (iv) spray-drying, (v) ionic gelation, and (vi) polyelectrolyte complexation [17,18]. Ionic gelation and polyelectrolyte complexation are the widest-used techniques for the development of NP systems, due to their ease of formation and absence of organic solvents. However, one of the main problems associated with the development of this type of NP is related to the type and concentration of the crosslinking agent [19,20]. Most of the crosslinking agents used for the production of chitosan NPs are molecular polyanions, such as tripolyphosphate [21,22,23], phytic acid [24,25] and macromolecular polyanions, such as poly(lactic-co-glycolic acid) [26], sodium alginate [27,28,29], and gum arabic [30,31,32,33,34]. To date, there are few reports dealing with the creation of chitosan nanoparticles with anionic polymers derived from maleic anhydrides, which are biocompatible and water soluble, and have a well-defined structure and hydrophilic or hydrophobic character that can be feasibly varied, allowing the encapsulation of different compounds of pharmaceutical interest [35]. Therefore, the goal of this study is to produce and characterize MTX-chitosan NPs using several novel polyanion polymers, such as the sodium and potassium salts of poly(maleic acid-*alt*-octadecene) [36,37,38,39] and poly(maleic acid-*alt*-ethylene) [40], named as PAM-18 and PAM-2, respectively. Further, high-intensity ultrasound, along with polyelectrolyte complexation, was used to generate nanoparticulate systems that have an improved MTX release profile.

## 2. Experimental

### 2.1. Materials

Methotrexate (lot LRAB9958) was purchased from Sigma Aldrich Co (Missouri, United States). Sodium hydroxide (lot B1315798639), acetic acid (lot K41575763) and potassium hydroxide (lot BO484233) were obtained from Merck (Darmstadt, Germany). Deacetylated chitosan, previously synthesized and characterized [41,42], was provided by the Laboratory of Design and Development of Drug and Cosmetics Products from University of Antioquia (Medellín, Colombia). This polymer was used as received. The anionic polyelectrolytes corresponding to the sodium and potassium salts of poly(maleic acid-*alt*-ethylene) (PAM-2Na or PAM-2K) and poly(maleic acid-*alt*-octadecene) (PAM-18Na or PAM-18K), previously synthesized and characterized [40], were provided by the Laboratory of Design and Formulation of Chemical Products from Icesi University (Cali, Colombia). Such anionic polymers were utilized as received.

### 2.2. Preparation of NP Systems

**Figure 1:**
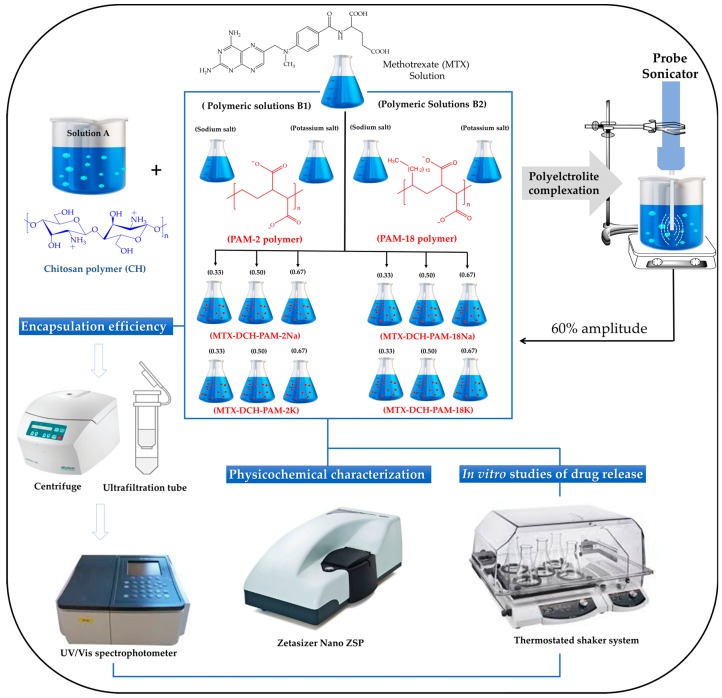
Schematic of the formation of chitosan–polyanion nanoparticles unloaded and loaded with methotrexate, using high-intensity ultrasound. The sodium and potassium counterions are not shown in the scheme.

The formation process of chitosan–polyanion nanoparticles unloaded and loaded with methotrexate, using high-intensity ultrasound, is depicted in Figure 1.

Briefly, a 3 mg/mL deacetylated chitosan solution (in 1% acetic acid, *v/v*) was prepared at a pH of 3.5 and labelled as solution A. At the same time, one solution of MTX (10 mg/mL in 0.5 N NaOH) and four aqueous polymer solutions (0.5 mg/mL) were prepared and named as solutions B1 (PAM-2Na and PAM-2K) and solutions B2 (PAM-18Na and PAM-18K). Subsequently, different mixtures were made by pouring 0.5 mL of MTX solution onto 10.0 mL, 15.0 mL or 20.0 mL of each solution B. Then, such mixtures (MTX solution + Solutions B) were poured into 5.0 mL of solution A, under constant magnetic stirring at 800 rpm and 25 ± 1 °C for 10 min. Therefore, the resulting chitosan:anionic polymer ratios were 1:0.33, 1:0.50 and 1:0.67, respectively. These mixtures were then left under constant stirring for another 10 min in order to generate the MTX-DCH-PAM-2 and MTX-DCH-PAM-18 complexes by ionic association.

Once the polyelectrolyte complexes were formed, they were processed by high intensity ultrasound, forming a nanoparticulate system. Briefly, a 4.0 mL aliquot of each polyelectrolyte complex dispersion was taken and sonicated using an ultrasonic probe (Q125 Sonicator coupled with standard probe of 3.2 mm, Melville, New York, NY, USA), operated at 30 s pulses followed by 30 s of rest for a total time of 5 min. An energy intensity of 1878 W corresponding to a 60% amplitude was utilized. Likewise, blank nanoparticles were also created following the same procedure, except for the absence of MTX, in solution “B”.

### 2.3. Physicochemical Characterization of NPs

#### 2.3.1. Particle Size, Polydispersity Index (PDI) and Zeta Potential Analyses

These analyses were determined using a Zetasizer nano ZSP (Malvern Instrument, Worcestershire, UK) equipped with a red He/Ne laser (633 nm). Particle size was measured using dynamic light scattering (DLS) with a scattered angle of 173° at 20 °C, and a quartz flow cell (ZEN0023), whereas the zeta potential was measured using a disposable folded capillary cell (DTS1070). This instrument reports the particle size as the z-average diameter, and PDI ranging from 0 to 1 corresponding to a monodisperse and very broad distributions, respectively. All the nanoparticles were dispersed in ultra-pure water, employing a ~1:100 *v/v* dilution factor. All measurements were performed in triplicate and reported as the mean ± standard deviation.

#### 2.3.2. Encapsulation Efficiency (EE)

The encapsulation efficiency of MTX was assessed by employing the ultrafiltration/centrifugation technique. An aliquot of each NP suspension was poured into an ultrafiltration tube (VWR, Modified PES 10 kDa, 500 µL) and centrifuged (MIKRO 185, Hettich Lab Technology, Tuttlingen, Germany) at 10,000 rpm for 6 min. Subsequently, 200 µL of the filtrate solution was taken and mixed with 800 µL of a mixture of water:1% (*v/v*) acetic acid (80:20). The absorbance of the resulting mixture was measured on a UV/Vis spectrophotometer (UV-1800, Shimadzu, Milton Keynes, UK) at 305 nm. The amount of MTX was determined by interpolation from a calibration curve built at concentrations of 1.5, 2.2, 3.7, 7.3, 11.0, 14.7, 18.3 and 22.0 µg/mL using a mixture of water/1% (*v/v*) acetic acid (80:20) as solvent. The quantity of MTX loaded inside the nanoparticles was calculated using the following expression:

(1) $$\mathrm{EE}=\frac{Q_{t}-Q_{s}}{Q_{t}}*100\%$$

where *Q_t_* and *Q_s_* correspond to the total amount of methotrexate and the amount of methotrexate found in the filtrate, respectively.

### 2.4. Physical Stability of the Nanoparticulate Systems

The physical stability of the MTX-loaded nanoparticulate systems was evaluated in stability chambers maintained at 4 °C and 40 °C for 5 days. Approximately, 2 mL of the nanoparticulate suspensions were stored, and the physicochemical parameters, such as particle size, polydispersity index and zeta potential were measured at the initial and final stage of the experiment, as previously described in Section 2.3.

### 2.5. In Vitro Release Studies of Methotrexate

The in vitro release of MTX was conducted on a thermostated shaker system (Unimax 1010, Heidolph Instruments, Schwalbach, Germany) operated at 37 °C and 50 rpm. A 4.0 mL aliquot of the nanoparticle suspension was placed in a dialysis bag (cut-off 12 kDa) and immersed in 80 mL of phosphate buffer (pH = 7.4) with 0.15 M ionic strength. Subsequently, 1.0 mL aliquots were periodically taken for MTX determination and replaced by the same volume of fresh medium. The concentration of MTX was determined by UV spectrophotometry at 305 nm by interpolation from a calibration curve built at concentrations of 2.0, 4.0, 6.0, 8.0, 10.0, 15.0, and 20.0 µg/mL. Release profile data were reported as the mean residence time (MRT) within the nanoparticles [43]. Further, the release mechanism of MTX from the nanoparticles was evaluated by fitting the release data to several heuristic models, including order one [44,45], Korschmeyer–Peppas [46,47], Peppas–Sahlin [48] and Higuchi [49] to establish if diffusion is driven by the concentration gradient and/or swelling, or whether erosion of the NPs is responsible for the MTX release. In addition, comparison between the release profiles was conducted using the *f_2_* similarity factor [50].

### 2.6. Statistical Analysis

Data were tabulated and analyzed using the Minitab^®^ v. 17 software (Minitab^®^ Inc., State College, PA, USA). Statistical comparisons were made employing the ANOVA test, where the effect of the polyanion type and polymer mass ratios on the particles size, PDI, zeta potential and encapsulation efficiency were evaluated. The Tukey *post-hoc* test was utilized to determine significant differences between the independent groups. A confidence level of 95% was adopted and data were expressed as mean ± standard deviation.

## 3. Results and Discussion

### 3.1. Production and Characterization of Nanoparticulate Systems

The formation of micro and nano-aggregates was due to the electrostatic interactions between deacetylated chitosan and polymer salts derived from maleic anhydride, resulting in a milky suspension when the system was stirred [51]. In this context, the deacetylated chitosan in acidic media acquired a positive charge due to the protonation of the primary amine groups in the backbone polymer. On the contrary, the PAM-2 and PAM-18Na salts tend to generate structures with a net negative charge. Thus, when the attractive interaction of both polyelectrolytes is set into place, several electrostatic interactions are generated in the CH chains, leading to a net polyelectrolytic complexation. Further, high-intensity ultrasound rendered nanometric systems by forming acoustic cavities in the aqueous media, resulting in shock waves and large shear stress, which are responsible for the dispersive and coagulation phenomena [52].

**Figure 2:**
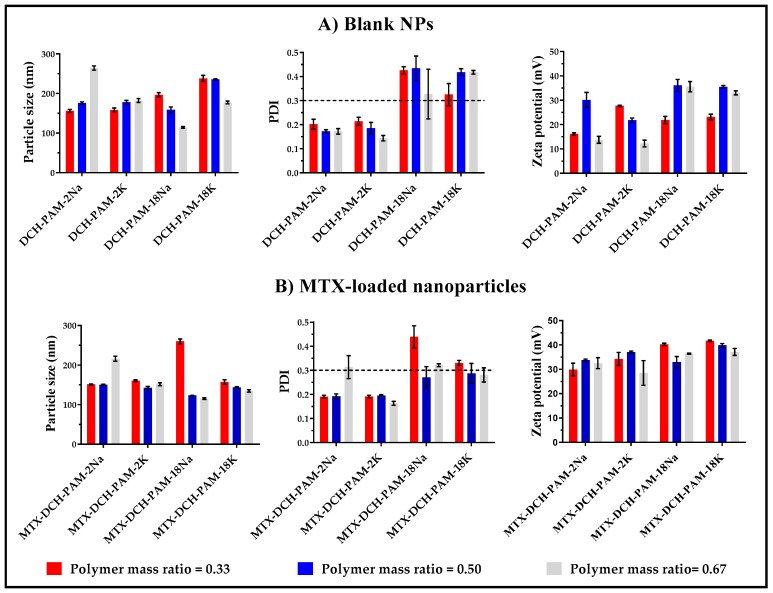
Particle size, polydispersity index-PDI and zeta potential characterization of (**A**) blank NPs and (**B**) MTX-loaded NPs in different mass ratios with chitosan.

The results from the physicochemical characterization of the blank nanoparticles and methotrexate-loaded nanoparticles (i.e., particle size, PDI and zeta potential) obtained by high-intensity ultrasound are shown in Figure 2.

#### 3.1.1. Particle Size

The results of the statistical analysis in Table S1 showed that the sizes of the blank NPs were significantly affected by the polyanion type (*p*-value *=* 0.004), whereas the polymer mass ratio showed no effect (*p*-value *=* 0.985). On the contrary, in MTX-loaded NPs, size was not affected by the polyanion type (*p =* 0.441), whereas the polymer mass ratio did affect this parameter (*p =* 0.023). In order to understand these findings, a more detailed discussion is provided in the following section.

The size of the blank NPs formed with both polyanions (PAM-2Na and PAM-2K) increased with the polymer mass ratio, ranging from 150 nm to 270 nm and 150 nm to 190 nm for PAM-2Na and PAM-2K, respectively (Figure 2A). This effect was more pronounced (264.3 ± 5.3 nm) for PAM-2Na NPs, having a polymer mass ratio of 0.67. These large sizes are produced as a result of the electrostatic attraction between deacetylated chitosan and PAM-2 salts, which had a prominent molecular weight (~100 kDa). Further, an increase in the chitosan:PAM-2Na mass ratio enhanced the particle size of MTX-loaded PAM-2Na NPs (MTX-DCH-PAM-2Na). However, this behavior was not verified on MTX-loaded PAM-2K NPs (MTX-DCH-PAM-2K). This is explained on the basis of counterion type, where the sodium ion is less strongly bounded to the polyanion agent as compared to the potassium ion, resulting in a major migration of PAM-2Na towards DCH, forming polyelectrolyte complexes [53,54,55,56,57] of larger sizes. Moreover, the inclusion of MTX generated a slight reduction in particle size. In this scenario, MTX-DCH-PAM-2Na and MTX-DCH-PAM-2K NPs exhibited particle sizes ranging from 149 nm to 225 nm and from 130 nm to 163 nm, respectively (Figure 2B). This effect can be explained by the formation of more reticulated structures driven by ion-dipole interactions and hydrogen bonds between polar moieties of MTX such as carboxylic acids, amides and amines [58] with the polyelectrolyte complexes of DCH and PAM-2 salts.

On the other hand, a reduction in particle size with an increasing chitosan:PAM-18 ratio was verified for blank NPs formed with sodium or potassium PAM-18 salts (DCH-PAM-18Na NPs and DCH-PAM-18K NPs, respectively). In this case, DCH-PAM-18K NPs exhibited sizes ranging from 174.0 nm to 243.3 nm, whereas DCH-PAM-18Na NPs showed sizes from 112.6 nm to 202.4 nm (Figure 2A). Moreover, the addition of MTX triggered the reduction of particle size, except for MTX-DCH-PAM-18Na NPs where the size increased from 238.1 ± 7.9 nm to 259.9 ± 6.1 nm (Figure 2B). This change is explained by the counterion type as mentioned previously. Likewise, PAM-18 is an amphiphilic polymer and in aqueous media it acquires a random configuration, resulting in polymer aggregates that associate more tightly to DCH by weak interactions, resulting in more compact structures [59,60,61].

#### 3.1.2. Polydispersity

Concerning the statistical analysis, the PDI of blank NPs (*p*-value = 0.000) and MTX-loaded NPs (*p*-value *=* 0.000) was significantly affected by the polyanion type. On the other hand, PAM-18 rendered NPs with non-homogeneous sizes (PDI > 0.3), whereas nanoparticles formed with PAM-2 were essentially monodisperse (PDI < 0.3). In addition, the polymer mass ratio did not affect this parameter.

The DCH-PAM-2Na and DCH-PAM-2K NPs showed PDI values ranging from 0.162 to 0.216, and 0.136 to 0.233, respectively. Furthermore, the inclusion of MTX and the increase in the chitosan:PAM-2 ratio caused a slight reduction in PDI values (Figure 2A,B), except for MTX-DCH-PAM-2Na NPs at a 0.67 ratio, whose PDI values increased from 0.172 ± 0.012 to 0.314 ± 0.048. These results agreed with those of the particle size. On the other hand, the DCH-PAM-18Na and DCH-PAM-18K NPs rendered PDI values ranging from 0.230 to 0.481 and 0.298 to 0.431, respectively. Moreover, MTX-DCH-PAM-18Na NPs had PDI values ranging from 0.238 to 0.485 and, MTX-DCH-PAM-18K NPs showed PDI values from 0.247 to 0.339. The reduction in PDI values with the addition of MTX is explained by the amphiphilic nature of PAM-18 salts as mentioned previously.

#### 3.1.3. Zeta Potential

According to the statistical analysis, the zeta potential of blank NPs was affected by the polyanion type (*p*-value = 0.001) and the polymer mass ratio (*p*-value = 0.024). In fact, the highest zeta potential values were obtained with the PAM-18 salts, due to the ability of this polyanion to interact with DCH by electrostatic and weak interactions (between lateral hydrocarbon chain) caused by its intrinsic amphiphilic nature. Conversely, the zeta potential of MTX-loaded nanoparticles was only affected by the polyanion type (*p*-value = 0.000). Further, the zeta potential of MTX-loaded NPs was higher than blank NPs, suggesting that MTX triggered major weak interactions such as London and Van Der Waals forces.

In this scenario, DCH-PAM-2 and MTX-DCH-PAM-2 NPs exhibited zeta values ranging from +10.7 mV to +33.6 mV, and from +10.7 mV to +33.6 mV, respectively (Figure 2A,B). Moreover, blank NPs achieved with PAM-18 showed zeta potential values ranging from +20.3 mV to +38.2 mV. Once methotrexate was added, these values increased from +30.3 mV to +42.1 mV (Figure 2A,B). In all cases, the zeta potential values were positive due to the excess of DCH, which was 1.5 to 3.0-fold higher than polyanions.

#### 3.1.4. Encapsulation Efficiency

**Figure 3:**
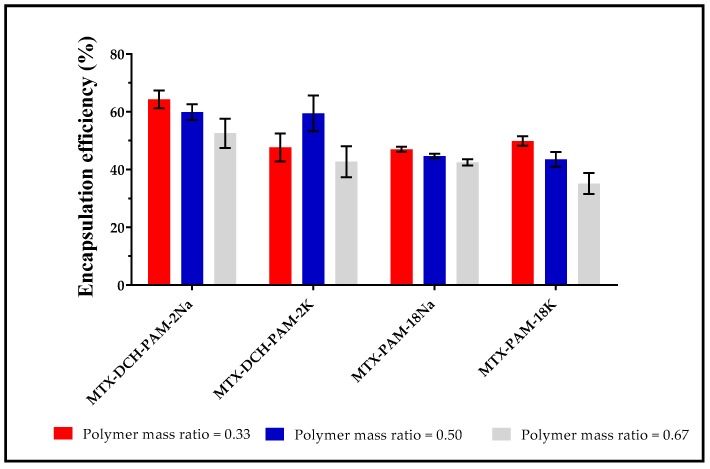
Encapsulation efficiency for nanosystems formed between deacetylated chitosan and several polyanions.

Regarding the statistical analysis, the encapsulation efficiency was affected by the polyanion type (*p*-value = 0.000). Thus, the highest MTX encapsulation was obtained with PAM-2Na, followed by PAM-2K, PAM-18Na and PAM-18K. Likewise, the polymer mass ratio affected this parameter (*p*-value = 0.014), due to a reduction in encapsulation as observed with the increment of polymer mass ratio. This phenomenon is explained by the loss of MTX loading capability of the NPs as a result of particle size reduction. Therefore, these nanometric systems can trap between 32% and 66% MTX as illustrated in Figure 3.

In the case of NPs obtained with DCH and PAM-2, it is assumed that MTX is trapped into a reticulated structure. Moreover, the PAM-18 NPs can associate MTX in the same way as PAM-2 NPs. Additionally, interaction of MTX with the lateral hydrocarbon chain of PAM-18 NPs (hydrophobic domains) occurred. This result is very interesting, because it would be expected that DCH-PAM-18 NPs should have a higher MTX encapsulation efficiency due to their two interaction mechanisms. Therefore, such a result suggests that MTX tends to interact better with DCH-PAM-2 NPs due to its greater hydrophilic character.

### 3.2. Physical Stability of the Nanoparticulate Systems

**Figure 4:**
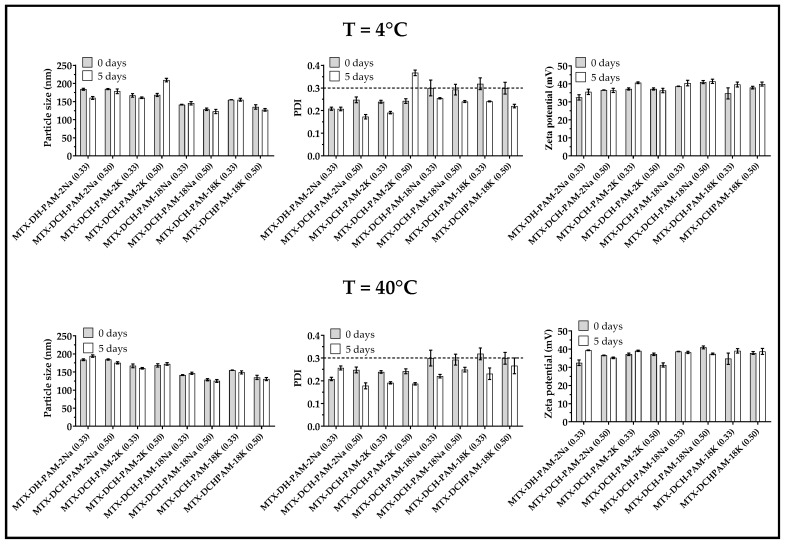
Stress studies conducted at 4 °C and 40 °C for MTX-loaded nanoparticulate systems.

Results from the physical stability study conducted at 4 °C and 40 °C are depicted in Figure 4. Particle size did not change significantly upon storage, except for MTX-DCH-PAM-2K systems where an increment in particle size and PDI in samples stored at 4 °C was evident. This phenomenon is explained by particle aggregation and free polymer chain interactions with the particle network, causing a synergistic rearrangement of intermolecular entanglements, and swelling phenomena by the incorporation of the surrounding solvent [62]. Likewise, these changes were observed at lower polymer mass ratios, where there was a major content of chitosan with respect to the polyanion agent, favoring the formation of larger nanoparticles [63].

Further, the PDI decreased for other systems, suggesting that a reduction in temperature increased the degree of polyelectrolyte complexation between deacetylated chitosan and the anionic polymer, generating a more homogeneous size. Some authors reported that chitosan NPs crosslinked with fucoidan maintained their physicochemical properties up to six weeks [64]. Moreover, slight changes in zeta potential were observed at this temperature.

Additionally, all the NPs were stable at 40 °C and only slight changes in particle size and PDI were observed. This is attributed to the rearrangement of the polymer chains leading to a higher polyelectrolyte complexation effect. Moreover, the zeta potential values were ~30 mV, indicating good electrostatic repulsive forces [65]. Therefore, such results confirmed that these nanoparticulate systems are physically stable over time and thus they did not show any significant change in the physicochemical properties studied.

### 3.3. In Vitro Release Studies of Methotrexate

**Figure 5:**
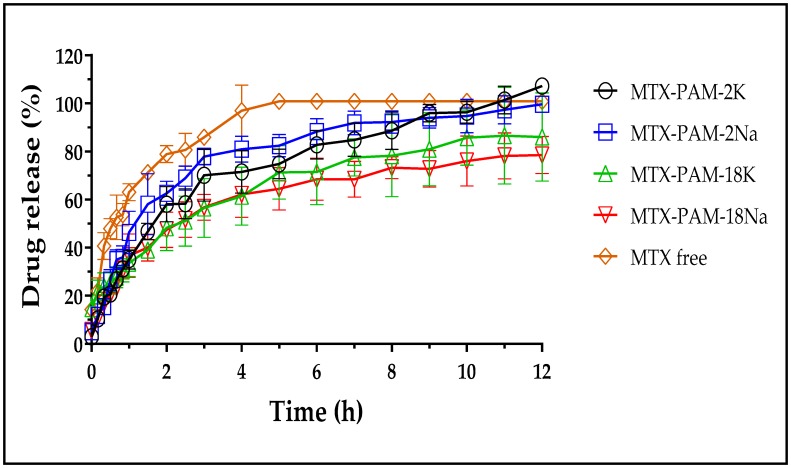
Methotrexate release profiles from nanoparticulate systems at 37 °C, 50 rpm and pH 7.4.

The release profiles of MTX from NPs obtained at a polymer mass ratio of 0.50, due to have the lowest particle size and high encapsulation efficiency according to the statistical analysis, are depicted in Figure 5. It is evident that methotrexate release reduced once incorporated into the nanoparticulate systems. For instance, sodium MTX was completely released, whereas the NP_S_ release was 70–100% at 12 h, indicating a controlled release.

Moreover, Table 1 lists the mean residence time (MRT) values of free MTX and polymer nanoparticulated systems. These values were higher for the NPs than free MTX, indicating a decline in drug release rates.

On the other hand, comparison of release profiles was conducted based on the *f_2_* values and the results are summarized in Table 2. Values between 50 and 100 indicate similarity between the release curves, whereas lower values are considered significantly different. Results indicate a dual similarity between MTX-PAM-18K and MTX-PAM-18Na, and between MTX-PAM-2Na and MTX-PAM-18Na, release profiles. Moreover, MTX-PAM-2K and MTX-PAM-2Na showed different release profiles. Likewise, the release curve from PAM-2 and PAM-18 salts were different (*f_2_* values ranged from 43.3 to 47.9). These findings agreed with those obtained from the physicochemical characterization of NPs suggesting different mechanisms for nanoparticle formation.

In order to elucidate the possible drug release mechanism from the polymeric NPs, a kinetic analysis was performed using diverse heuristic models. The resulting parameters from the models that showed the best data fit are summarized in Table 3.

In particular, free MTX showed a good fit to the Higuchi model, suggesting that the release was driven by Fickian drug diffusion in that media. Moreover, the polymer nanoparticulate systems showed a burst release between 2.0% and 15.0%. This phenomenon can be attributed to the release of MTX located in the outer part of the NPs, where the medium penetrates initially. Moreover, MTX-PAM-2K, MTX-PAM-2Na and MTX-PAM-18K NPs showed a good fit to the Korshmeyer–Peppas model (>0.9576). In this scenario, “n” values ranging from 0.43 to 0.85 indicate an anomalous diffusion of MTX from NPs where the drug release was controlled by the relaxation of the polymer chains and erosion of the NPs [66,67]. Further, MTX-PAM-18Na NPs data fitted the Peppas–Sahlin model well, indicating that MTX release was driven by diffusion and polymer relaxation. In this case, the diffusion mechanism was predominant according to the *k_d_* and *k_r_* values [68].

**Figure 6:**
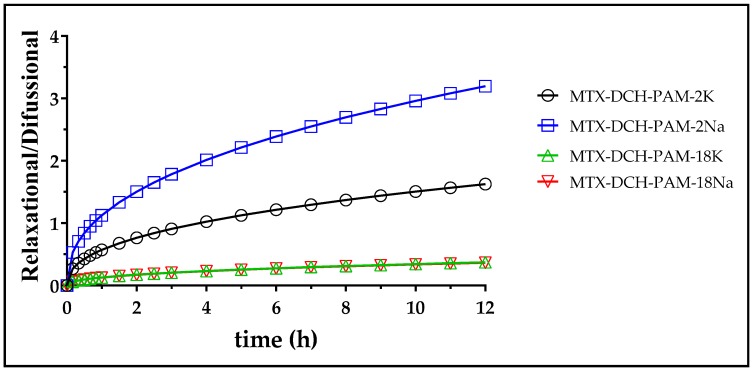
Relaxational to diffusional ratio as a function of time for methotrexate release from nanoparticles at a pH of 7.4.

Even though the Peppas–Sahlin model showed a lower degree of fit to the data than the Korshmeyer–Peppas model, its derived form was utilized for understanding the magnitude and contribution of the relaxational and diffusive mechanisms responsible for MTX release from NPs [69]. The ratio of the relaxational to the diffusional behavior (R/F) of NPs is depicted in Figure 6. Except for MTX-PAM-2Na, the diffusional component prevailed for most NPs [70]. Further, NPs produced with PAM-18 showed the largest diffusional contribution to MTX release, showing the largest drug retention, whereas NPs produced with PAM-2 anions showed more relaxation/gelling contribution, easing drug release.

## 4. Conclusions

The production of novel chitosan nanoparticles was assessed by high-intensity ultrasound-assisted polyelectrolyte complexation with different anionic crosslinking polymers, rendering a particle size smaller than 280 nm. Size was affected by the amount and type of crosslinking used. However, the nanoparticles generated with PAM-18 were not homogeneous in size. The zeta potential values were between +22.7 to +42.1, suggesting adequate electrostatic repulsion that prevented particle aggregation. Likewise, nanoparticulate systems exhibited encapsulation efficiencies ranging from 32% to 66%. These nanoparticles released methotrexate by an anomalous mechanism. Most nanoparticles showed a predominant diffusional release mechanism, whereas PAM-2Na NPs exhibited a prevalent relaxational mechanism. In this form, these NPs can be used as a novel carrier for MTX release by the intravenous route and establish a starting point for studies of these nanocarriers on drug delivery of pharmaceutically active compounds.

## Figures and Tables

**Table 1:** Mean residence time (MRT) values of MTX released from polymer nanoparticulate systems at pH = 7.4.

Polymer	Free MTX	MTX-PAM-2K	MTX-PAM-2Na	MTX-PAM-18K	MTX-PAM-18Na
MRT	0.8	1.2	1.1	1.3	1.3

Values calculated at 5 h.

**Table 2:** Similarity factor (*f*_2_) of polymeric nanoparticulate systems at a pH of 7.4.

Release Profiles	*f*_2_ Factor
MTX-PAM-2K-MTX-PAM-2Na	43.3
MTX-PAM-18K- MTX-PAM-18Na	55.1
MTX-PAM-2Na- MTX-PAM-18K	47.9
MTX-PAM-2Na- MTX-PAM-18Na	65.5
MTX-PAM-2K- MTX-PAM-18K	47.2
MTX-PAM-2K- MTX-PAM-18Na	46.6

**Table 3:** Fitting parameters of several kinetic models for MTX release.

Polymer	Order One $LogQ_{t}=LogQ_{0}-\frac{k_{1}}{2.303}t$		Higuchi $Q_{t}=k_{H}t^{1/2}$		Korshmeyer-Peppas with Burst $\frac{M_{t}}{M_{\infty }}=k_{r}t^{n}+b$				Peppas-Sahlin $\frac{M_{t}}{M_{\infty }}=k_{d}t^{0.43}+k_{r}t^{0.85}$		
	k	r^2^	k	r^2^	K	n	b	r^2^	k_d_	k_r_	r^2^
**Free MTX**	0.72	0.9479	40.8	0.9688	-	-	-	-	-	-	-
**MTX-PAM-2K**	0.30	0.9669	30.5	0.9748	0.33	0.66	0.02	0.9818	0.19	0.16	0.9803
**MTX-PAM-2Na**	0.35	0.9136	28.5	0.9171	0.40	0.85	0.04	0.9745	0.08	0.35	0.9687
**MTX-PAM-18K**	0.16	0.9821	23.3	0.9824	0.18	0.76	0.15	0.9916	0.36	0.00	0.8344
**MTX-PAM-18Na**	0.12	0.9262	22.3	0.9482	0.28	0.61	0.01	0.9576	0.24	0.08	0.9704

## Supplementary Materials

The following are available online at https://www.mdpi.com/1424-8247/13/1/11/s1, Table S1: Results from the *post-hoc* Tukey test assessing the effect of polyanion type and polymer mass ratio on the physicochemical characteristics of chitosan-polyanion nanoparticles (i.e., particle size, PDI, zeta potential and encapsulation efficiency).

## Abbreviations

MTX	Methotrexate
DCH	Deacetylated chitosan polymer
CH	Chitosan polymer
PAM-2Na	Sodium salt of poly(maleic acid-*alt*-ethylene)
PAM-2K	Potassium salt of poly(maleic acid-*alt*-ethylene)
PAM-18Na	Sodium salt of poly(maleic acid-*alt*-octadecene)
PAM-18K	Potassium poly(maleic acid-*alt*-octadecene)
NPs	Nanoparticles
