# Supplementary material for: Production and Characterization of Chitosan–Polyanion Nanoparticles by Polyelectrolyte Complexation Assisted by High-Intensity Sonication for the Modified Release of Methotrexate
Source: Pharmaceuticals (Basel). 2020 Jan 8;13(1):11. doi: 10.3390/ph13010011 (PMC7168905; doi:10.3390/ph13010011)
Supplement: Supplementary file 1 [file pharmaceuticals-13-00011-s001.pdf]

Table S1. Results from the *post-hoc* Tukey test assessing the effect of polyanion type and polyanion: chitosan ratios on the physicochemical characteristics of chitosan-polyanion nanoparticles (i.e., particle size, PDI, zeta potential and encapsulation efficiency).

Factor	**Methotrexate-unloaded nanoparticles**			**Methotrexate-loaded nanoparticles**		
	**Particle size (nm)**			**Particle size (nm)**		
	level	average	group	level	average	group
Polyanion type	PAM-18K	217.0	A	PAM-2Na	172.5	A
	PAM-2Na	198.7	A B	PAM-18Na	166.1	A
	PAM-2K	172.9	A B	PAM-2K	151.6	A
	PAM-18Na	156.5	B	PAM-18K	145.2	A
PA: CH ratio	0.33	187.3	A	0.33	182.2	A
	0.50	186.9	A	0.67	154.4	A B
	0.67	184.6	A	0.50	139.9	B
	**Polydispersity**			**Polydispersity**		
**Factor**	level	average	group	level	average	group
Polyanion type	PAM-18Na	0.396	A	PAM-18Na	0.344	A
	PAM-18K	0.387	A	PAM-18K	0.300	A B
	PAM-2Na	0.182	B	PM-2Na	0.232	B C
	PAM-2K	0.181	B	PAM-2K	0.183	C
PA: CH ratio	0.50	0.303	A	0.33	0.288	A
	0.33	0.292	A	0.67	0.270	A
	0.67	0.265	A	0.50	0.237	A
	**Zeta potential (mV)**			**Zeta potential (mV)**		
**Factor**	level	average	group	level	average	group
Polyanion type	PAM-18Na	+31.2	A	PAM-18K	39.6	A
	PAM-18K	+30.5	A	PAM-18Na	36.6	A B
	PAM-2K	+20.6	B	PAM-2K	33.3	B C
	PAM-2Na	+20.0	B	PAM-2Na	32.1	C
PA: CH ratio	0.50	+30.9	A	0.33	36.5	A
	0.67	+23.7	A B	0.50	35.9	A
	0.33	+22.2	B	0.67	33.7	A
	**Encapsulation efficiency (%)**			**Encapsulation efficiency (%)**		
**Factor**	level	average	group	level	average	group
Polyanion type	-	-	- - - -	PAM-2Na	58.9	A
	-	-	- - - -	PAM-2K	49.9	B
	-	-	- - - -	PAM-18Na	44.7	B
	-	-	- - - -	PAM-18K	42.8	B
PA: CH ratio	-	-	- - - -	0.33	52.2	A
	-	-	- - - -	0.50	51.9	A
	-	-	- - - -	0.67	43.2	B

The means that do not share a letter are significantly different.
